# The Role of CyaY in Iron Sulfur Cluster Assembly on the *E. coli* IscU Scaffold Protein

**DOI:** 10.1371/journal.pone.0021992

**Published:** 2011-07-20

**Authors:** Clara Iannuzzi, Salvatore Adinolfi, Barry D. Howes, Ricardo Garcia-Serres, Martin Clémancey, Jean-Marc Latour, Giulietta Smulevich, Annalisa Pastore

**Affiliations:** 1 Medical Research Council National Institute for Medical Research, London, United Kingdom; 2 Dipartimento di Chimica “Ugo Schiff”, Università di Firenze, Sesto Fiorentino, Firenze, Italy; 3 Commissariat pour l'Energie Atomique, iRTSV/LCBM, Grenoble, France; 4 CNRS, UMR 5249, Grenoble, France; 5 Université Joseph Fourier, Grenoble, France; Emory University School of Medicine, United States of America

## Abstract

Progress in understanding the mechanism underlying the enzymatic formation of iron-sulfur clusters is difficult since it involves a complex reaction and a multi-component system. By exploiting different spectroscopies, we characterize the effect on the enzymatic kinetics of cluster formation of CyaY, the bacterial ortholog of frataxin, on cluster formation on the scaffold protein IscU. Frataxin/CyaY is a highly conserved protein implicated in an incurable ataxia in humans. Previous studies had suggested a role of CyaY as an inhibitor of iron sulfur cluster formation. Similar studies on the eukaryotic proteins have however suggested for frataxin a role as an activator. Our studies independently confirm that CyaY slows down the reaction and shed new light onto the mechanism by which CyaY works. We observe that the presence of CyaY does not alter the relative ratio between [2Fe2S]^2+^ and [4Fe4S]^2+^ but directly affects enzymatic activity.

## Introduction

Iron sulfur (Fe-S) clusters are essential prosthetic groups formed by assemblies of iron and sulfide centres [1]–[5]. Best known for their role in the oxidation-reduction reactions of the mitochondrial electron transport chain, Fe-S clusters have many other functions, which include generation of radicals, gene expression regulation and biosynthesis of sulfur derivatives [4], [6].

While obtainable *in vitro* from inorganic sources of iron and sulfur, *in vivo* cluster assembly is achieved by dedicated machineries, which in bacteria are grouped in well-defined operons [7], [8]. Proteins from the *nif* and *suf* operons are active in specific conditions/organisms, whereas the gene products of the *isc* operons are the most general source of Fe-S clusters in bacteria and have highly conserved orthologs in eukaryotes [9]. The *isc* machinery is centered on a desulfurase (IscS or Nfs1 in prokaryotes and eukaryotes respectively) which catalyzes conversion of cysteine into alanine and inorganic sulfide ([10], [11], reviewed in [2]). The cluster is transiently assembled on the scaffold protein IscU/Isu which releases it to other acceptors. The process involves complex multiprotein interactions involving chaperones, ferredoxin and other ancillary proteins whose role is still unclear.

One such partner is frataxin, a small acidic protein whose reduced expression causes the neurodegenerative Friedreich's ataxia by impairing Fe-S cluster biogenesis and inducing iron accumulation [12]. As a consequence of its direct involvement in human disease, frataxin has attracted considerable attention but its function remains a matter of debate. The possibility that frataxin acts as a regulator of Fe-S biogenesis has emerged recently [13], [14]: using optical techniques, we have shown that CyaY, the bacterial frataxin ortholog, binds to IscS and slows down the kinetics of Fe-S cluster formation on IscU [13]. A role as a regulator was also proposed in two more recent studies on human frataxin, where however the authors reported a frataxin-induced activation [15], [16]. This important discrepancy calls for the use of complementary methods which could allow a more thorough understanding of the effect of CyaY/frataxin on the kinetics of cluster assembly on IscU.

Herein, we have carried out further analysis of the properties of CyaY on the enzymatic activity of IscS. Our study had different specific objectives. First, we wanted to confirm from an independent perspective our previous observations, based on absorbance and circular dichroism (CD) spectroscopies [13], that CyaY slows down Fe-S cluster assembly in IscU. Second, we were seeking new hints to understand the mechanism by which CyaY operates. We reasoned that our previous data could have an ambiguous interpretation as the techniques used can not inherently discriminate whether CyaY has an effect on the inhibition of a particular type of cluster ([2Fe-2S]^2+^ vs [4Fe-4S]^2+^) or is of a more global nature (i.e. it blocks cluster formation altogether). Finally, we wanted to understand further if CyaY operates at the level of the cysteine-to-alanine conversion or in cluster formation. To achieve this aim, we need to obtain a better and semi-quantitative description of the activity of bacterial IscS which could then be compared with that published for the human protein [15].

We have used a combination of techniques, which include resonance Raman (RR) and Mössbauer spectroscopy complemented by electronic absorption, NMR and amino acid analysis. RR and Mössbauer spectroscopies have been widely used to characterize [2Fe-2S]^2+^ clusters bound to the IscU and NifU scaffold proteins and to study their transformation into [4Fe-4S]^2+^ clusters under reductive conditions [11], [17], [18]. Additionally, both techniques can unambiguously discriminate between different types of iron complexes and iron sulfur clusters, enabling a more reliable analysis of the Fe-S biogenesis process [19], [20]. Interestingly, they have usually been applied to final purified species or their mixtures, whereas we have used them here to follow the course of Fe-S cluster assembly. To our knowledge, there is only one other study, carried out on NifU in which Mössbauer spectroscopy was used to follow cluster formation kinetics [17]. NMR and amino acid analysis allowed us to obtain a detailed and complementary description of the enzymatic activity and to dissect the role of the different components.

Taken together, our data confirm that CyaY acts as an inhibitor of the iron sulfur cluster assembly rates that operates by directly inhibiting the enzyme activity, and provide further information about the mechanism by which regulation occurs.

## Results

### Characterisation of the IscU-bound clusters by absorption, RR and Mössbauer spectroscopies

**Figure 1:**
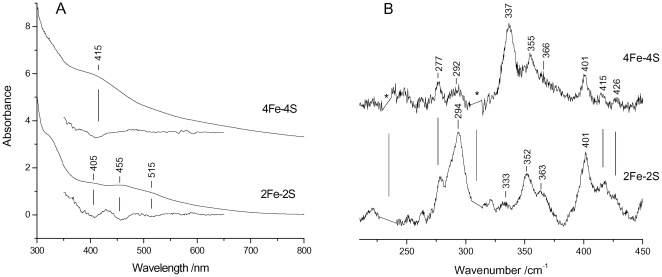
RR and electronic absorption spectra of Fe-S clusters assembled on *E. coli* IscU. (A) Electronic absorption spectra in both normal and second derivative presentations of [2Fe-2S]^2+^ and [4Fe-4S]^2+^ clusters, and (B) RR spectra of almost pure [2Fe-2S]^2+^ and [4Fe-4S]^2+^ clusters. RR experimental conditions: excitation wavelength 413.1 nm, spectral resolution 1 cm^−1^, laser power at the sample 55 mW; ([2Fe-2S]^2+^) average of eleven spectra with 15 min integration time; ([4Fe-4S]^2+^) average of nine spectra with 10 min integration time. The asterisks indicate laser plasma lines that have been removed.

The evolution of Fe-S cluster formation has extensively been described using electronic absorption spectroscopy [11], [21]. Electronic absorption spectra of Fe-S proteins provide clear evidence for the presence of Fe-S clusters allowing discrimination between [2Fe-2S]^2+^ and [4Fe-4S]^2+^ species. Similar to other Fe-S proteins, the 300–800 nm spectrum of the [2Fe-2S]^2+^ cluster formed on IscU is characterised by bands at 320, 405, and 455 nm and a shoulder at about 515 nm, as highlighted by the second derivative (D^2^) spectrum (**Figure 1A**, bottom). As the reaction proceeds, or upon reduction, the absorbance spectrum changes and the formation of the [4Fe-4S]^2+^ cluster can be readily identified on the basis of the single band detected at 415 nm (**Figure 1A**, top) [11], [18]. It should however be noted that the band at 415–420 nm overlaps the absorption bands of other iron complexes; we have for instance shown before that iron-loaded IscS and CyaY have bands around the same wavelength in the absence of any other component [13].

RR gives a more specific picture of the same time course. RR spectra recorded for excitation in resonance with the S→Fe charge transfer band in the visible region selectively enhance both the bridging (b) and terminal (t) Fe-S stretching vibrations (**Figure 1B**). Since the frequencies of these bands depend on the type of Fe-S cluster, RR spectra of Fe-S proteins in the Fe-S stretching region (200–450 cm^−1^) provide a sensitive means for identifying Fe-S clusters and discriminating between clusters such as [2Fe-2S]^2+^ and [4Fe-4S]^2+^ [19]. In particular, the intense bands at 294, 352 and 401 cm^−1^ of the [2Fe-2S]^2+^ cluster are assigned to the B_3u_^t^, A_g_^t^, and A_g_^b^ modes, slightly upshifted compared to those of ferredoxins [22], and the characteristic intense band at 337 cm^−1^ of the [4Fe-4S]^2+^ cluster is assigned to the Fe-S^b^ totally symmetric breathing mode [22]–[24].

Mössbauer spectroscopy is highly suited for studying iron-containing proteins. In particular, it has been successfully used to follow the assembly of [2Fe-2S]^2+^ clusters in IscU and NifU scaffold proteins and their transformation into [4Fe-4S]^2+^ clusters under reductive conditions [11], [17], [18]. The Mössbauer spectroscopic signatures of [2Fe-2S]^2+^ and [4Fe-4S]^2+^ clusters are markedly distinctive. The resonance intensities are strictly proportional to the relative amounts, thus making it possible to quantitatively follow the cluster assembly process. The [2Fe-2S]^2+^ cluster ligation of *A. vinelandii* IscU comprises three conserved cysteines and a histidine, making the two Fe^3+^ sites non-equivalent [11]. Consequently, they appear in the 4.2 K Mössbauer spectrum as two distinct quadrupole doublets, with different isomer shifts and quadrupole splittings (δ_1_ = 0.27(3) and δ_2_ = 0.32(3) mm/s; ΔE_Q1_ = 0.66(5) and ΔE_Q2_ = 0.94(5)). By contrast, the [4Fe-4S]^2+^ cluster at 4.2 K appears as two quadrupole doublets with very similar isomer shifts (δ_1_ = 0.46(2) and δ_2_ = 0.45(2) mm/s, but distinct quadrupole splittings ΔE_Q1_ = 1.07(4) and ΔE_Q2_ = 1.23(4) mm/s), due to two valence delocalised Fe^2+^Fe^3+^ pairs [18].

### The effect of CyaY on the Mössbauer spectrum of IscU

**Figure 2:**
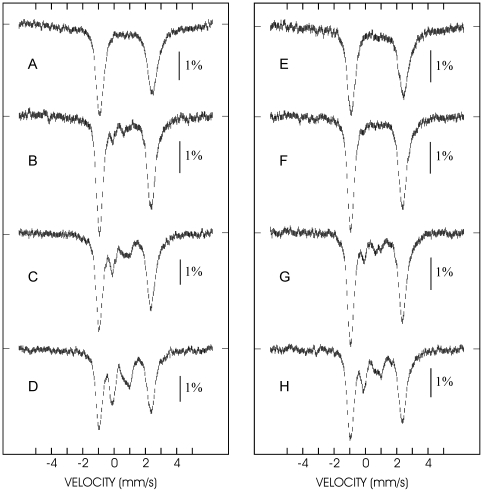
Mössbauer spectra of the FeS cluster assembly reaction. The spectra were recorded in the absence (left panel, spectra A (top) – D (bottom)) and in the presence (right panel, spectra E (top) – H (bottom)) of CyaY. The spectra were recorded at 4.2 K and with a magnetic field of 60 mT applied parallel to the γ ray.

**Figure 3:**
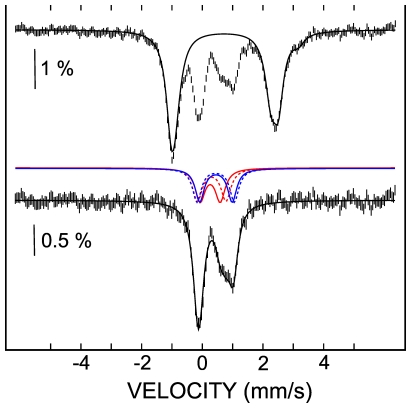
Analysis of the Mössbauer spectrum. The spectrum D of figure 3 is deconvoluted. The spectrum was recorded in the absence of CyaY at 4.2 K and with a magnetic field of 60 mT applied parallel to the γ ray. The top spectrum illustrates the deconvolution of the contribution of the ferrous ions. The bottom spectrum was obtained by subtracting the simulated contribution of the ferrous ions (black solid line in top spectrum) from the experimental spectrum. The remaining contribution results from a mixture of a [2Fe-2S]^2+^ cluster (red lines) and a [4Fe-4S]^2+^ cluster (blue lines).

**Figure 4:**
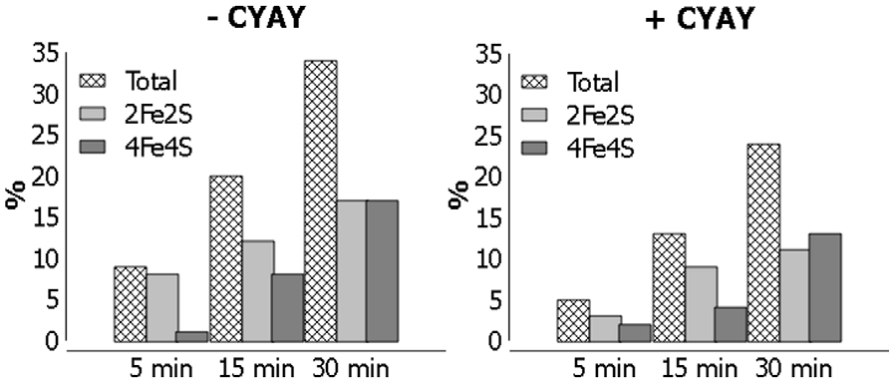
Time dependence of FeS cluster formation deduced from the Mössbauer experiments. The individual contributions of the different complexes are estimated at each time point (5, 15 and 30 min) in the absence (left) and in the presence (right) of CyaY.

The time dependence of Fe-S cluster formation was followed by Mössbauer spectroscopy upon freezing ^57^Fe-enriched samples at different time points after initiation of the reaction. Three separate time course experiments were monitored with duration times in the range 30 to 120 min. A representative experiment recorded over a 0–30 min period in the absence (**Figures 2A–D**) and in the presence (**Figures 2E–H**) of CyaY is presented. In the absence of CyaY, the initial spectrum (**Figure 2A**) consists of a broad asymmetric doublet that is associated with Fe^II^ species, mostly tetrahedral ferrous thiolates [18], [25]. The spectrum recorded after 5 min (**Figure 2B**) shows the appearance of a weak doublet in the velocity region −0.5–1 mm/s. As time elapses (**Figures 2C,D**), the left absorption corresponding to the low energy peak of this doublet increases but its right counterpart both grows in intensity and undergoes some deformation owing to the development of a new absorption at ca. 1 mm/s. This behaviour is consistent with the successive appearance of at least two species. The spectra in **Figures 2B–D** were simulated as mixtures of the ferrous components present in **Figure 2A** and the [2Fe-2S]^2+^ and [4Fe-4S]^2+^ clusters, that could be assigned according to the literature parameters cited above. In all cases excellent fits were obtained. The spectrum associated with the Fe-S clusters after subtraction of the ferrous component from the spectrum in **Figure 2D** and its deconvolution shows that (i) initially the [2Fe-2S]^2+^ cluster grows faster so that it dominates at 5 min (8% of total iron vs 1% for the [4Fe-4S]^2+^ clusters) and at 15 min (12% vs 8%) but (ii) then the [4Fe-4S]^2+^ grows faster so that at 30 min the two have the same weight (17% of total iron vs 17% for the [2Fe-2S]^2+^ clusters) (**Figure 3**). The time dependence of the formation of the clusters is illustrated in **Figure 4**.

Examination of **Figures 2E–H** indicates that in the presence of CyaY the same species are formed but the appearance of the Fe-S clusters is markedly slower. The spectra were analysed using the above-mentioned parameters for the ferrous species and the clusters. In the absence of CyaY, the [2Fe-2S]^2+^ cluster dominates the mixture at short times (3% vs 2% at 5 min and 9% vs 4% at 15 min) but is progressively overwhelmed by the [4Fe-4S]^2+^ cluster as the reaction proceeds (11% of total iron belonging to [2Fe-2S] clusters vs 13% belonging to [4Fe-4S] clusters at 30 min). Comparison of the two series of data (**Figure 4**) reveals that the main effect of CyaY on the reaction is to slow down the reaction: after 30 min only 25% of clusters are formed in the presence of CyaY, while 35% are formed in its absence. On the other hand, CyaY does not seem to have a significant effect on the relative abundance of the two clusters.

The reproducibility of these conclusions was confirmed by two additional independent experiments undertaken over extended time courses up to 2 h (data not shown).

### The effect of CyaY on the RR spectrum of IscU

**Figure 5:**
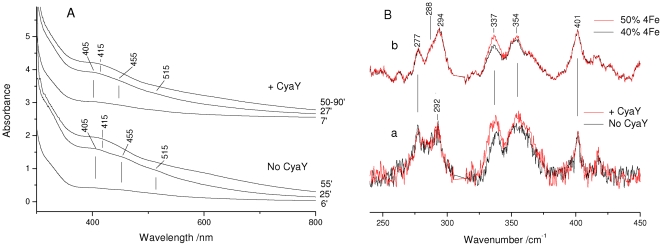
Kinetics of cluster formation. (A) Electronic absorption spectra obtained during the time course of Fe-S synthesis on *E. coli* IscU at the times indicated. (B) (a) Superposition of the RR spectra obtained during the time course of Fe-S synthesis in the time interval 30–60 min (no CyaY, black) and 30–90 min (+CyaY, red). (b) Superposition of the RR spectra for admixtures of the pure species shown in Figure 1: 50% [4Fe-4S]^2+^ /50% [2Fe-2S]^2+^ (red) and 40% [4Fe-4S]^2+^ /60% [2Fe-2S]^2+^ (black). RR experimental conditions: excitation wavelength 413.1 nm, spectral resolution 1 cm^−1^, laser power at the sample 50 mW; (no CyaY) average of 5 spectra with 10 min integration time; (+CyaY) average of 10 spectra with 10 min integration time. The spectra are normalised on the 294 cm^−1^ band of the [2Fe-2S]^2+^ cluster. The asterisk indicates a laser plasma line which has been removed.

We then exploited the electronic absorption and RR spectral properties of the nearly pure forms of the [2Fe-2S]^2+^ and [4Fe-4S]^2+^ clusters assembled on IscU described above (**Figure 1**) to follow in parallel by both techniques the kinetics of Fe-S clusters formation in the absence and in the presence of CyaY at room temperature. The electronic absorption spectra observed at corresponding times in the absence and in the presence of CyaY are very similar (**Figure 5A**). In agreement with the Mössbauer spectra, in both sets of spectra, the variations indicate a progressive increase in the proportion of the [4Fe-4S]^2+^ clusters at the expense of [2Fe-2S]^2+^. In the presence of CyaY, the time course of Fe-S formation is extended, indicating that the rate of Fe-S synthesis is reduced. In both cases, the variations observed in the electronic absorption spectra suggest that the conversion of [2Fe-2S]^2+^ to [4Fe-4S]^2+^ is already at an advanced stage after ca. 25 min, coincident with the beginning of the RR experiment. However, the RR spectra could not be analysed prior to 30 min because of their poor signal-to-noise ratio.

The RR spectra with excitation at 413.1 nm were recorded in the time interval 30–60 min (in the absence of CyaY) and 30–90 min (in the presence of CyaY) after sample preparation (**Figure 5B**). At the end of the indicated time intervals, both the absorbance and RR signals weakened considerably and the solution became dark-grey in agreement with the visible precipitation of iron sulfide. Unlike the electronic absorption (at room temperature) and Mossbauer (at low temperature), the RR spectra recorded during this time course in the presence and absence of CyaY do not display remarkable changes in the relative band intensities, between [2Fe-2S]^2+^ and [4Fe-4S]^2+^. Superposition of the RR spectra in the presence and in the absence of CyaY (**Figure 2B**, bottom) shows only a minor relative increase of the [4Fe-4S]^2+^ band at 337 cm^−1^, confirming that the presence of CyaY does not significantly favour an increase in the proportion of [4Fe-4S]^2+^.

Hence, consistent with the Mossbauer experiments, UV-Vis and RR spectra at room temperature indicate that the presence of CyaY lengthens the time course of Fe-S cluster synthesis.

### Quantification of the enzymatic activity of IscS

Finally, having confirmed our previous observations, we wanted to characterize further the enzymatic activity of IscS and understand which process is inhibited by the presence of CyaY. The spectroscopic techniques used here or previously described for the CyaY/IscS system follow cluster formation. This is however the final product of at least three distinct processes: i) enzymatic conversion of cysteine into alanine with production of persulfide, which is the direct enzymatic reaction; ii) transfer of this group to IscU, which presumably involves the movement of a flexible loop from the active site to IscU [8]; iii) cluster assembly on IscU. It is therefore important to decouple the different steps. We used two independent techniques to test directly the CyaY effect on IscS enzymatic activity.

**Figure 6:**
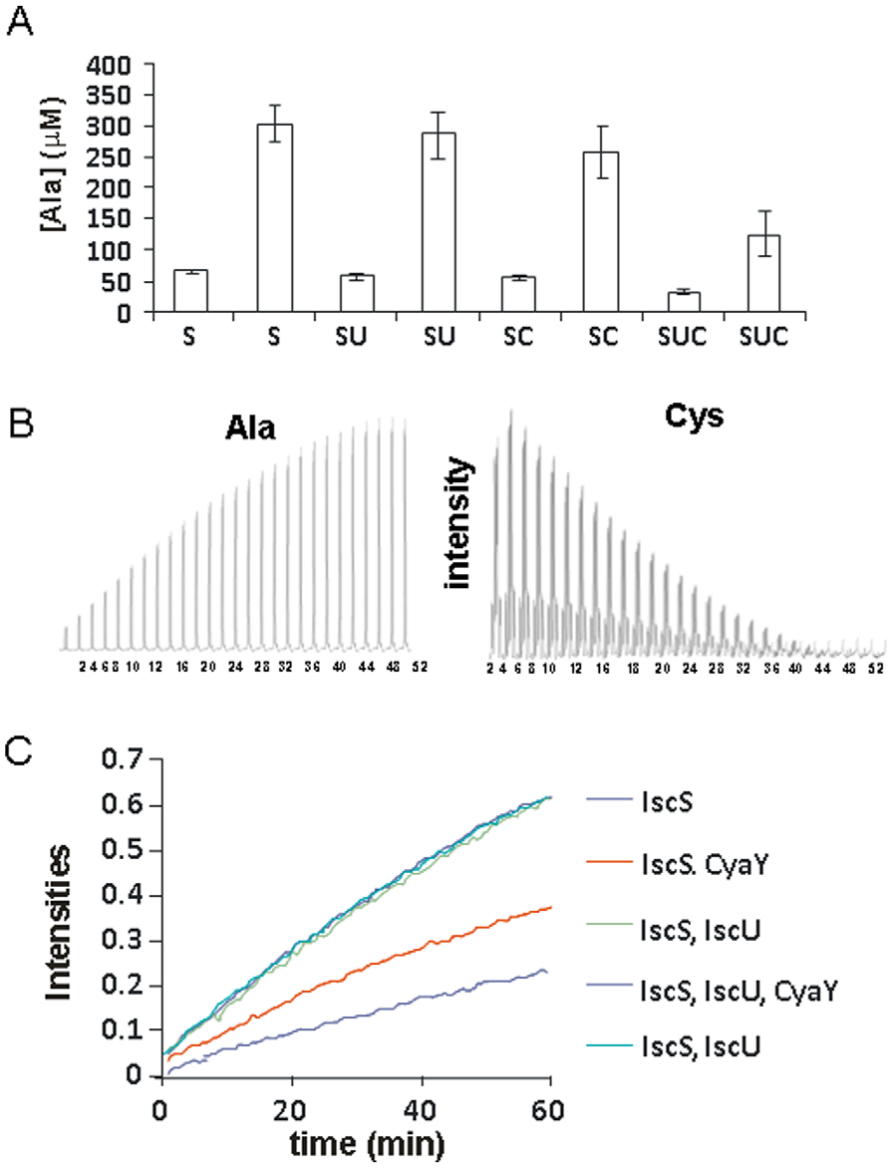
Influence of CyaY on the enzymatic activity of IscS. **A**) Dosage of the alanine formed at different times. Each measurement is reported twice and corresponds to 5 and 20 min after starting the reaction. S, SU and SUC indicate different mixtures of IscS, IscU and CyaY. **B**) Kinetics of cysteine consumption with concomitant alanine formation. Peak intensities of the b-protons of alanine (left) and cysteine are reported as a function of time. **C**) Same as in B) but plotting the results of experiments recorded in the presence of different mixtures of IscS, IscU and CyaY. The two curves in the presence of IscU (which superpose perfectly) correspond to two independent kinetics performed with 12 hours interval to check for reproducibility.

We first tested alanine formation by amino-acid analysis. Although not completely accurate, because of difficulties in detecting cysteine and because of the presence of the protein background, this technique can provide a semi-quantitative estimate of the concentrations of alanine formed at given time points (we chose to test them at 5 and 20 minutes). The enzymatic reaction was started by addition of cysteine and stopped by addition of trichloroacetic acid (TCA) (20%). We observed that addition of IscU does not affect the reaction. Addition of CyaY, and even more of CyaY together with IscU, has a noticeable effect on alanine production (**Figure 6A**).

These data were complemented by one-dimensional ^1^H NMR experiments. By this technique, it is possible to follow the decay of well-isolated cysteine resonances (e.g. Hβ protons) with the concomitant progressive appearance of the alanine (Hβ protons) resonances (**Figure 6B**). This method is efficient and highly accurate and allows direct and continuous substrate detection. Under the assay conditions, the conversion kinetics reached a plateau in ca. 20–40 minutes. The assay was repeated in the presence and absence of CyaY, IscU and their co-presence. When IscU was added no effect was observed on the enzymatic activity (**Figure 6**C). Addition of CyaY showed a clear decrease of IscS initial rate. This decrease was even more accentuated when both CyaY and IscU were added to the solution.

These results demonstrate that the inhibitory effect of CyaY is exerted already at the level of the enzyme activity.

## Discussion

The function of frataxin has been debated for years. The only consensus that has emerged is that frataxin has a link with Fe-S assembly, based both on genetic and biochemical evidence [26], [27]. Within this context, quite different hypotheses have suggested that frataxin behaves as a ferritin-like storage protein or an iron chaperone [28]–[30]. The view emerging more recently is that frataxin is a regulator of Fe-S cluster assembly through interactions with IscS and IscU, the two main components of the *isc* machinery [13]. However, independent studies have reported an inhibitory role for bacterial frataxin but activation for the human orthologue [13], [15], [16].

We have studied here the effect of bacterial frataxin on Fe-S cluster formation on IscU. To obtain a deeper insight into the role of the bacterial CyaY in the process of Fe-S cluster formation we have used different techniques to check different aspects of the influence of CyaY on the cluster formation reaction *in vitro*. First, we have reconsidered our previous results using two complementary techniques, RR and Mössbauer spectroscopies. Both techniques have clear advantages with respect to electronic absorption, the technique most commonly used for the study of cluster formation kinetics [11], [21]. Albeit powerful, this technique has the limitation of detecting at approximately the same wavelength all iron complexes, making it very difficult to distinguish them unequivocally. To partially circumvent this problem and avoid being misled to wrong conclusions, it is essential to record the whole spectrum at each time point rather than following the evolution of the absorption signal at only one wavelength, both to be sure of the type of complex observed and to minimize background effects. This is unfortunately not always done.

Another technique that has been used to follow Fe-S formation is near-UV CD, which avoids the complications due to the overlapping absorption spectra of other iron-bound components and is very sensitive to the cluster nuclearity. However, CD detects almost exclusively [2Fe-2S]^2+^ clusters, giving sparse information on [4Fe-4S]^2+^ clusters with the exception of 2[4Fe-4S]^2+^ bacterial ferredoxins [31]. The CD spectrum of [4Fe-4S]^2+^ loaded IscU, for instance, has weak bands which can barely be distinguished from the baseline, at least for moderate IscU concentrations.

A major advantage of RR and Mössbauer is that they both allow identification of different types of clusters through distinctive signatures which also allows the clusters to be distinguished from unspecific iron complexes. Characterization of IscU bound [2Fe-2S]^2+^ and [4Fe-4S]^2+^ clusters by these techniques have been reported, providing valuable spectroscopic reference markers [11], [18].

The data shown here confirm unequivocally our previous results and show a clear-cut reduction of the rates of Fe-S assembly in the presence of CyaY [13]. Under our experimental conditions, CyaY operates as a negative regulator *in vitro*. The presence of CyaY does not appear to alter significantly the mutual distribution of the two types of clusters. CyaY, therefore, operates in a global manner, slowing the formation of both types of clusters.

We have then analysed in more detail whether CyaY affects directly enzymatic activity, persulfide transfer or cluster formation on IscU. We observe that desulphurase activity is greatly affected by the presence of CyaY and even more so when present together with IscU (CyaY/IscU), whereas it is not affected by the presence of IscU alone. While these results lead to different conclusions from those previously obtained [13]–[14], this is the first time that we could measure with high accuracy the effect of IscU and CyaY directly on the production kinetics of alanine formation in a continuous way and without the need of coupling enzymatic activity with other reactions. Also in this assay we observe that CyaY slows down the reaction. A way to rationalize these observations could be that CyaY interferes with catalytic activity by masking or modifying the active site. We have in fact previously shown that CyaY binds to IscS with a 1∶1 stoichiometry in a pocket very close to the active site but well distinct from the binding region of IscU [14]. CyaY could thus induce a conformational change indirectly or physically block the enzymatic activity. Interference would be greater when both CyaY and IscU are bound, given that their co-presence increases their mutual affinities for IscS as previously shown by bilayer interferometry and isothermal titration calorimetry studies [14].

How can we reconcile our results with those obtained in the same *in vitro* assay for human frataxin which seems to cause enzyme activation rather than inhibition? An easy way to explain the difference is by assuming that different orthologues might have different specificities. This would however be in strong contrast both with the high conservation throughout evolution of the sequences of both frataxin and its partners, and with the at least partial rescuing effect that CyaY has on frataxin depleted eukaryotic cells [32]. A more convincing hypothesis is to accept that, since frataxin takes part in a complex network of competing interactions, the *in vitro* behaviour of the eukaryotic and prokaryotic proteins could differ and we are not comparing them under similar conditions or in comparable states. In the eukaryotic system, for instance, human and yeast frataxins have been used only in combination with a complex of Nfs1 with the Isd11 protein, which is absent in prokaryotes [33], [34]. Isd11 seems to stabilize recombinant Nfs1 and protect it from aggregation and/or misfolding but could also alter its conformational state and its affinity for other partners. Another crucial question concerns the conditions under which the system operates in the cell and, in particular, the relative concentrations of the different components. Only by answering these questions and by finding ways to validate *in vivo* our *in vitro* results we may hope to reach some understanding of the fascinating but difficult question of the cellular role of frataxin.

## Materials and Methods

### Protein production

Recombinant CyaY, IscS and IscU from *E. coli* were prepared as previously described [35], [36]. In short, they were produced as fusion proteins with a His-tagged GST and purified by affinity chromatography using Ni-NTA agarose gel (QIAGEN). The collected proteins were cleaved overnight from GST by TEV protease and further purified by gel-filtration chromatography on a Superdex 75 26/60 column (GE Healthcare). All purification steps were carried out in the presence of 20 mM β-mercaptoethanol. Protein purity was checked by SDS-PAGE and by mass-spectrometry. Dithiothreitol (DTT), Fe^2+^ (as ferrous ammonium sulphate) and cysteine were purchased from Sigma. ^57^Fe-enriched Mohr's salt (>99%) for Mössbauer samples was purchased from Chemgas.

### Sample preparation for absorbance and RR measurements

Preparation of near pure [2Fe-2S]^2+^ and [4Fe-4S]^2+^ species was obtained by mixing, respectively, i) 10 mM DTT, 1 mM Fe^2+^, 20 µM IscS, 100 µM CyaY, 5 mM cysteine to 1 mM IscU; and ii) 5 mM DTT, 2 mM Fe^2+^, 20 µM IscS, 100 µM CyaY, 5 mM cysteine to 1 mM IscU. The values refer to final concentrations. The samples for the time course experiments of Fe-S cluster formation were prepared using the same concentrations as in i) except for the use of 10 µM IscS. The choice of the relative concentration has been discussed in detail previously [13]. In short, the concentration of IscU, which is the final cluster acceptor in the assay, was set to a value which would allow easy measurement of the cluster according to the technique in use. IscS was set to a low concentration to avoid even minimal contributions of unspecific iron-thiolate polysulfides bound to IscS [31]. The IscS/IscU molar ratio also determines the time scale in which the kinetics are completed [11]. Likewise, we used small concentrations of CyaY (typically 100 µM). The presence of the anti-oxidant DTT is necessary to provide reducing equivalents required for cluster generation and to regenerate the prosthetic group pyridoxal phosphate (PLP) [21]. The relative concentrations of the components of the reaction mixture were chosen as in previous studies, but the total concentrations were increased to allow for the lower sensitivity of RR and Mössbauer spectroscopies [13], [14]. The individual components were added in the order as written.

### Parallel absorbance and Raman spectroscopies

Electronic absorption and RR spectra were measured in parallel at room temperature using anaerobically sealed samples in a 5 mm NMR tube. Absorption spectra were registered with a double-beam Cary 5 spectrophotometer (Varian, Palo Alto, CA) and a 600 nm/min scan rate. Second derivative D^2^ spectra (LabCalc, Galactic Industries, Salem, NH) were obtained using the Savitzky-Golay method with 15 data points. No changes in the wavelength or in the bandwidth were observed when the number of points was increased or decreased.

The RR spectra were obtained by excitation with the 413.1 nm line of a Kr^+^ laser (Coherent, Innova 300 C, Santa Clara, CA) and the 457.9 nm line of an Ar^+^ laser (Coherent, Innova 90/5, Santa Clara, CA). Previous RR studies of other Fe-S synthesis systems have been carried out at low temperature [18] to improve spectral resolution and prevent laser-induced sample degradation. In our case, the moderate fluorescence observed at room temperature was considerably enhanced at low temperature compromising spectral quality and making low temperature experiments impracticable. Nevertheless, the spectral resolution of our spectra is comparable to that of published Fe-S cluster spectra. In addition, the spectral variations with time were reasonably slow enabling a number of successive spectra to be added to improve the final quality. Backscattered light from a slowly rotating NMR tube was collected and focused into a triple spectrometer (consisting of two Acton Research SpectraPro 2300i and a SpectraPro 2500i in the final stage with a 3600 grooves/mm grating) working in the subtractive mode, equipped with a liquid nitrogen-cooled CCD detector. The spectral resolution of the RR spectra as reported in the figure captions was calculated theoretically on the basis of the optical properties of the spectrometer. However, for the moderately broad experimental RR bands observed in the present study (ca. 10 cm^−1^), the effective spectral resolution will be in general lower. The RR spectra were calibrated with CCl_4_ and dimethyl sulfoxide as standards to an accuracy of ±1 cm^−1^ for intense isolated bands.

The RR experiment ended when both the electronic absorption and RR signals weakened considerably and the solution became dark-grey from the visible precipitation of iron sulfide. It is worth mentioning that, at the end of the reaction, we observe iron precipitation in the whole range of iron concentrations used in the present and in previous studies (5 mM–1.2 mM) [13], [14]. This occurs because, in the absence of a final acceptor, the cluster on IscU is very unstable and falls apart easily in the presence of even traces of oxygen. Precipitation is not observed in the Mössbauer experiments which do not reveal the presence of any significant amount of iron sulphide. This may affect the optical and RR experiments, but not the Mössbauer measurements since the samples are frozen and likely more protected from oxygen.

### Sample preparation for Mössbauer spectroscopy

A first series of samples for Mössbauer analyses was prepared by mixing anaerobically the following individual components in the given order to obtain the final concentrations of 1 mM IscU, 10 µM IscS, 5 mM DTT, 1.2 mM ^57^Fe^2+^ and 5 mM cysteine. The samples were frozen in liquid nitrogen at different times (0, 5, 15, 30, 60 or 120 min) after adding cysteine. A second series of experiments was run at the same freezing times on samples of identical compositions but in the presence of 50 µM CyaY. These experiments were carried out in three independent sets with overlapping time domains to assess the reproducibility of the observations. In addition, control experiments were performed for each of the three sets of experiments on various mixtures lacking a specific component:

Control 1: DTT 5 mM, ^57^Fe^2+^ 1.2 mM, CyaY 400 µM;

Control 2: IscU 1 mM, DTT 5 mM, ^57^Fe^2+^ 1.2 mM, Cys 5 mM;

Control 3: IscS 50 µM, DTT 5 mM, ^57^Fe^2+^ 1.2 mM;

Control 4: IscS 50 µM, DTT 5 mM, ^57^Fe^2+^ 1.2 mM, Cys 5 mM.

No Fe-S cluster formation was observed in any of these controls. All samples and controls for Mössbauer analyses were prepared within an inert atmosphere box with an oxygen content <2 ppm. They were frozen in liquid nitrogen within the box and kept at that temperature until measurement.

### Mössbauer spectroscopy

Mössbauer spectra were recorded at 4.2 K on a low-field Mössbauer spectrometer equipped with a Janis SVT-400 cryostat and weak field permanent magnets. The spectrometer was operated in a constant acceleration mode in transmission geometry. The isomer shifts are referenced against that of a room-temperature metallic iron foil. Analysis of the data was performed with the program WMOSS (WEB Research, Edina, MN, USA). Owing to the strong overlap of the contributions of the [2Fe-2S]^2+^ and [4Fe-4S]^2+^ clusters and their low content at the beginning of the reaction, great care was taken during the simulation of the spectra to reliably extract their relative abundances. In an initial phase, the contribution of the predominant ferrous species was fitted and then subtracted from the spectrum to be able to extract the cluster contributions. To avoid over-parameterisation of the system, parameters reported in the literature [18] were used for the isomer shifts and quadrupole splittings of the two clusters. The only variable parameters were for each cluster the linewidth and the abundance.

### Enzymatic assays

NMR ^1^H experiments were performed at 25°C on a VARIAN INOVA 600 MHz. Samples were prepared in 50 mM Tris-HCl pH 8.0, 250 mM NaCl, 5% D_2_O. The enzymatic reactions were prepared mixing IscS 12 µM, IscU 12 µM, CyaY 60 µM, DTT 3 mM and Cys 2 mM in an NMR tube. IscS or Cys were added to start the reaction. Monodimensional spectra were collected every two minutes. All the experiments were repeated at least twice on different batches of proteins.

Reaction samples were prepared by mixing IscS 12 µM, IscU 12 µM, CyaY 60 µM, DTT 3 mM. The enzymatic reactions were started adding 2 mM Cys. Aliquots at two time points (1 and 5 min or 5 and 20 min) were collected in duplicates. The reaction was quenched by adding 20% (w/v) trichloroacetic acid (TCA) and collected samples. After keeping the samples on ice for 10 min, we left them at −20°C overnight to allow slow protein precipitation. The solutions were centrifuged at 11,000 *g* for 5 min. The alanine content in the supernatant was quantified by amino acid analysis.
